# Exploring glycoside hydrolases and accessory proteins from wood decay fungi to enhance sugarcane bagasse saccharification

**DOI:** 10.1186/s13068-016-0525-y

**Published:** 2016-05-23

**Authors:** Fernanda Valadares, Thiago A. Gonçalves, Dayelle S. P. O. Gonçalves, Fernando Segato, Elisson Romanel, Adriane M. F. Milagres, Fabio M. Squina, André Ferraz

**Affiliations:** Departamento de Biotecnologia, Escola de Engenharia de Lorena, Universidade de São Paulo, Lorena, SP 12602-810 Brazil; Laboratório Nacional de Ciência & Tecnolologia do Bioetanol (CTBE), Centro Nacional de Pesquisa em Energia e Materiais (CNPEM), Campinas, SP 13083-970 Brazil; Departamento de Bioquímica, Instituto de Biologia (IB), Universidade Estadual de Campinas (UNICAMP), Campinas, SP 13083-862 Brazil

**Keywords:** Alkaline pretreatment, Biorefinery, Glycoside-hydrolases, Enzyme cocktails, Sugarcane, Wood decay fungi

## Abstract

**Background:**

Glycoside hydrolases (GHs) and accessory proteins are key components for efficient and cost-effective enzymatic hydrolysis of polysaccharides in modern, biochemically based biorefineries. Currently, commercialized GHs and accessory proteins are produced by ascomycetes. However, the role of wood decay basidiomycetes proteins in biomass saccharification has not been extensively pursued. Wood decay fungi degrade polysaccharides in highly lignified tissues in natural environments, and are a promising enzyme source for improving enzymatic cocktails that are designed for in vitro lignocellulose conversion.

**Results:**

GHs and accessory proteins were produced by representative brown- and white-rot fungi, *Laetiporus sulphureus* and *Pleurotus ostreatus*, respectively. Concentrated protein extracts were then used to amend commercial enzymatic cocktails for saccharification of alkaline-sulfite pretreated sugarcane bagasse. The main enzymatic activities found in the wood decay fungal protein extracts were attributed to endoglucanases, xylanases and β-glucosidases. Cellobiohydrolase (CBH) activities in the *L. sulphureus* and *P. ostreatus* extracts were low and nonexistent, respectively. The initial glucan conversion rates were boosted when the wood decay fungal proteins were used to replace half of the enzymes from the commercial cocktails. *L. sulphureus* proteins increased the glucan conversion levels, with values above those observed for the full load of commercial enzymes. Wood decay fungal proteins also enhanced the xylan conversion efficiency due to their high xylanase activities. Proteomic studies revealed 104 and 45 different proteins in the *P. ostreatus* and *L. sulphureus* extracts, respectively. The enhancement of the saccharification of alkaline-pretreated substrates by the modified enzymatic cocktails was attributed to the following protein families: GH5- and GH45-endoglucanases, GH3-β-glucosidases, and GH10-xylanases.

**Conclusions:**

The extracellular proteins produced by wood decay fungi provide useful tools to improve commercial enzyme cocktails that are currently used for the saccharification of alkaline-pretreated lignocellulosic substrates. The relevant proteins encompass multiple glycoside hydrolase families, including the GH5- and GH45-endoglucanases, GH3-β-glucosidases, and GH10-xylanases.

**Electronic supplementary material:**

The online version of this article (doi:10.1186/s13068-016-0525-y) contains supplementary material, which is available to authorized users.

## Background

The enzymatic hydrolysis of polysaccharides is a central process step in modern biorefineries [1]. Glycoside hydrolases (GHs) and accessory proteins are key components for this processing step [2–4]. Currently, most of the commercial enzyme preparations used in the hydrolysis of lignocellulosic materials comes from ascomycetes [4]. *Trichodermareesei* (*Hypocrea jecorina*), for example, produces several GHs that synergistically act in cellulose hydrolysis, and these GHs include two cellobiohydrolases (CBH—*Cel7A* and *Cel6A*) and four characterized endoglucanases (EG—*Cel5A*, *Cel7B*, *Cel12A* and *Cel45A*) [4, 5]. The synergistic actions of these enzymes depend on different, highly specific ratios between the individual proteins [4]. Several of these enzymes contain a cellulose binding module (CBM), which contributes to the adsorption of the enzyme to the substrate, enhancing the enzyme performance [6, 7]. β-glucosidases are also necessary components of the enzymatic cocktail that ensure the conversion of cellobiose into glucose [4, 8].

Accessory proteins found in ascomycetes include swollenins, which increase cellulases access to cellulose chains [9, 10]. Swollenins have only been detected in 11 species of ascomycetes to date according to UNIPROT. The *T. reesei* swollenin (SWOI) has an N-terminal CBM1 that is linked to a domain that shows homology with the plant expansin protein family [11]. Lytic polysaccharide mono-oxygenases (LPMOs, AA9) that oxidize glycoside linkages also contribute to initial cellulose disassembly [4, 12, 13]. Cellobiose dehydrogenases (CDHs) participate in the cellulose degradation system by oxidizing cellobiose and/or by acting as a redox enzymes, which integrates LPMOs actions [3]. CDHs can also reduce Fe^3+^ to Fe^2+^ and O_2_ to H_2_O_2_, which generates Fenton reagents during the in vivo degradation of lignocellulose [14].

Unlike the ascomycetes enzymes, the GHs and accessory proteins from basidiomycetes have not been extensively explored for cellulose saccharification. Wood decay basidiomycetes (white- and brown-rot fungi) are particularly important because they efficiently degrade polysaccharides in natural and controlled environments, even in highly lignified tissues [15, 16]. At least three EGs, three CBHs and seven β-glucosidases have been described in the *Phanerochaete chrysosporium* white-rot fungus [17, 18]. However, most brown-rot fungi are deficient in CBHs, which limits the enzymatic degradation of crystalline cellulose by this fungal group [18]. Nevertheless, some studies have suggested that processive EGs can compensate for CBH deficiencies in specific fungal species, including *Fomitopsis palustris* [19] and *Gloeophyllum trabeum* [20]. These EGs cleave cellulose internally, but also release soluble oligosaccharides and cellobiose.

Genome sequencing and data annotations from public databases have enabled our search for new enzymes in basidiomycetes [16–18, 21]. Enzymes from this fungal class offer a promising approach towards improving enzymatic cocktails designed for in vitro lignocellulose conversion. Studies aimed at evaluating the basidiomycetes secretomes have highlighted the diversity of the enzymes used by these species to degrade lignocellulose [22–28]. However, investigations of the enzymatic saccharification of lignocellulosic materials by GHs from basidiomycetes in vitro have been limited [29–31].

The aim of this study was to explore GHs and accessory proteins in representative white- and brown-rot fungi (*Pleurotus ostreatus* and *Laetiporus sulphureus*, respectively). Extracellular protein extracts were obtained from these species and used to amend commercial enzyme cocktails for polysaccharide hydrolysis in pretreated sugarcane bagasse. The protein composition of the fungal extracts was evaluated by LC–MS/MS. The results revealed several enzymes that enhanced cellulose and xylan hydrolysis in vitro.

## Results and discussion

### Endoglucanase activities in *Pleurotus ostreatus* and *Laetiporus sulphureus* cultures

Both fungal species were grown in submerged cultures containing different carbon sources, and a comparative evaluation revealed that high endoglucanase (EG) activities were evident in the media with sugarcane bagasse and carboxymethyl cellulose (CMC) (Table 1). The other tested carbon sources (listed in the “Methods” section) showed low or undetectable EG activities. *Pleurotus ostreatus* presented abundant mycelium on the medium surface when sugarcane bagasse was the only carbon source in the culture. This carbon source did not sustain the growth of *L. sulphureus*; only CMC promoted *L. sulphureus* growth in the submerged cultures. The time-course of enzymatic activities for these cultures indicated that *L. sulphureus* maximally produced EG activity at 0.90 IU/mL after 24 days in culture, when total extracellular protein level reached 62 mg/L. *Pleurotus ostreatus* cultures (grown on sugarcane bagasse) produced significant EG activity levels when the culturing time was longer (1.41 IU/mL after 42 days with an extracellular protein concentration of 190 mg/L). These activity and protein levels were similar to those reported for basidiomycetes [32] but were lower than the typical values measured for cellulolytic ascomycetes, such as *Trichoderma* species, when grown in optimal conditions [33]. Despite the studied basidiomycetes presented low levels of extracellular proteins and endoglucanase activities, the proteins produced by these fungal species could present high diversity, requiring additional characterization to explore their potential as supplementary proteins for biomass hydrolysis. Identified proteins can be overexpressed in more suitable hosts regarding enzyme secretion.

**Table 1:** Enzymatic activities of glycoside hydrolases detected in the *Laetiporus sulphureus* and *Pleurotus ostreatus* extracts and in reference commercial enzymatic preparations

Enzyme source	Hydrolytic activities in the protein extracts (IU mg^−1^ protein. In parenthesis are activity ratios setting β-glucosidase to the unit)					
	FPase	Endoglucanases	Cellobiohydrolases	β-Glucosidases	Xylanases	β-xylosidase
*L. sulphureus*	nd	2.2 (1.5)	0.11 (0.07)	1.5 (1.0)	43.1 (28.0)	0.15 (0.1)
*P. ostreatus*	nd	0.2 (1.0)	nd	0.2 (1.0)	0.4 (1.9)	0.04 (0.2)
*T. reesei*	0.42 (0.6)	5.1 (7.1)	0.94 (1.3)	0.7 (1.0)	4.5 (6.3)	0.27 (0.4)
*A. niger*	nd	nd	nd	12.1 (1.0)	40.6 (3.3)	0.13 (0.01)

*L. sulphureus* cultured in 2 % CMC for 24 days; *P. ostreatus* cultured on sugar cane bagasse for 42 days; commercial enzymes produced by *Trichoderma reesei* (*Hypocrea jecorina*) (SIGMA C2730), *Aspergillus niger* (SIGMA C6105)

*nd* Not detected

Based on these results, the carbon sources used to stimulate the secretomes of *P. ostreatus* and *L. sulphureus* were sugarcane bagasse and CMC, respectively. Protein concentrates were screened for GHs activities (Table 1). The highest activities were detected using the CMC (endoglucanases), birch wood xylan (xylanases) and 4-nitrophenyl β-d-glucopyranoside (β-glucosidases) substrates. Low activities were measured on crystalline cellulose (CBHs) in the *L. sulphureus* extract but were absent in the *P. ostreatus* extract. Little to no activity was observed on 4-nitrophenyl β-d-xylopyranoside (β-xylosidases). Because the filter paper conversion into reducing sugars did not reach 4 % after 60 min reaction, the filter paper activity was considered undetectable for all wood decay protein extracts [34].

The extracts described in Table 1 were used as a supplementary protein source for saccharification of pretreated sugarcane bagasse. The main source for the enzymes in these experiments was the complex mixture of cellulases produced by *T. reesei* (SIGMA C2730). The hydrolytic activities detected in these commercial extracts (Table 1) indicate that EG, CBH, and β-glucosidase were present at a ratio of 7.1:1.3:1. This extract is broadly used in enzymatic hydrolysis experiments that focus on lignocellulosic biomass conversion [4]. The low β-glucosidases proportion in this extract typically requires supplementation with *Aspergillus niger* enzymes to prevent cellulases inhibition by the cellobiose that accumulates during hydrolysis [8]. In the protein extracts recovered from the wood decay fungal cultures, the EG, CBH and β-glucosidase activity ratios were 1.5:0.07:1 and 1:0.00:1 for *L. sulphureus* and *P. ostreatus*, respectively. Besides the low CBH activity, a significant characteristic of each fungal extract was its xylanase activity, resulting in xylanase/EG ratios of 1.9:1 in *P. ostreatus* and 19:1 in *L. sulphureus*, compared to 0.9:1 for the *T. reesei* enzyme mixture.

### Enzymatic hydrolysis of pretreated sugarcane bagasse

The pretreatment of sugarcane bagasse was performed using a previously described alkaline-sulfite method [35]. The chemical composition and processing yields of the pretreated material indicated that suitable substrates were produced for enzymatic conversion of the polysaccharides. Specifically, 54 % of the original lignin and 41 % of the hemicellulose were removed during the pretreatment (Additional file 1: Table S1). Sulfonation of the residual lignin produces a highly hydrophilic substrate that promotes fiber cell wall swelling, which improves enzyme infiltration contributing to lowering the material recalcitrance [36, 37].

**Fig. 1:**
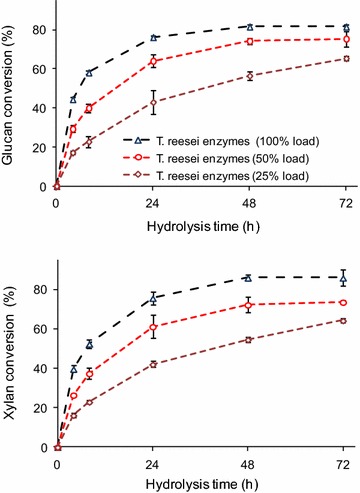
Glucan and xylan conversions of alkaline-sulfite pretreated sugarcane bagasse by enzymes produced by *T. reesei* and *A. niger*. Enzyme mixtures used in all of the experiments are shown in the inserted legends. A 100 % load indicates 10 FPU or 120 IU of endoglucanases from *T. reesei* (SIGMA C2730) +15 IU β-glucosidase from *A. niger* (SIGMA C6105)/g of substrate

The enzymatic saccharification of the pretreated sugarcane bagasse by the *T. reesei* enzymes was originally evaluated using three enzyme concentrations (Fig. 1). The experiments using various enzyme loadings indicated that the hydrolysis efficiency depended on the enzyme activity level in each reaction. The glucan conversion increased from 64 ± 1 to 82 ± 2 % when the enzyme loading increased from 2.5 to 10 FPU/g (Fig. 1). Similarly, the initial hydrolysis rates increased from 4.3 ± 0.3 to 11.1 ± 0.4 %/h for the same hydrolysis conditions (Table 2). The xylan conversion levels also depended on the enzyme loading and followed a similar pattern (Fig. 1; Table 2).

**Table 2:** Initial reaction rates during enzymatic hydrolysis of alkaline-sulfite pretreated sugarcane bagasse by several enzyme mixtures

Enzyme mixture used in the hydrolysis experiment	Initial hydrolysis rate (% h^−1^)*	
	Glucan	Xylan
*T. reesei* 100 % (10 FPU/g)	11.1 ± 0.4 (a)	9.9 ± 0.4 (a)
*T. reesei* 50 % (5 FPU/g)	7.3 ± 0.2 (b)	6.6 ± 0.2 (b)
*T. reesei* 25 % (2.5 FPU/g)	4.2 ± 0.3 (c)	4.0 ± 0.2 (c)
*T. reesei* (50 %) + *L. sulphureus* (50 %)	11.5 ± 0.3 (a)	11.2 ± 0.4 (ad)
*T. reesei* (50 %) + *P. ostreatus* (50 %)	11.8 ± 0.4 (a)	12.0 ± 0.3 (dd)
*T. reesei* (50 %) + *T. emersonii* (50 %)	9.0 ± 0.3 (d)	6.0 ± 0.2 (b)

In each column, the values with the same letters do not differ among themselves at significance level of 0.05

* Mean values were compared based on Tukey’s test (*p* < 0.01)

**Fig. 2:**
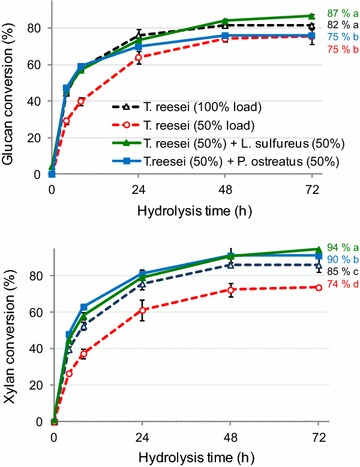
Glucan and xylan conversions of alkaline-sulfite pretreated sugarcane bagasse by enzymes from *T. reesei* and *A. niger* supplemented with protein extracts produced by *L. sulphureus* or *P. ostreatus*. The enzyme mixtures used for all the experiments are shown in the inserted legends. Polysaccharide conversions after the 72-h reactions followed by mean comparisons based on Tukey’s HSD (*p* < 0.05) are shown as inserted legends

To evaluate the wood decay fungal protein extracts for saccharification of pretreated sugarcane bagasse, we replaced 50 % of the original EG activity provided by the *T. reesei* enzymes with the EG activities from the wood decay fungal extracts (Fig. 2). The reference enzyme dosage was 10 FPU/g of substrate. This enzyme load corresponded to 120 IU of EG/g of substrate for the *T. reesei* commercial enzymes (Table 1). Because the wood decay fungal extracts were complex protein mixtures (Table 1; Additional file 2: Table S2), simultaneous activity variations were noted for several enzymes in the experiments. However, EG was used as a titer because it was the main cellulolytic activity detected in the wood decay fungal extracts.

The glucan conversion reached 82 ± 2 % after a 72 h of reaction with the *T. reesei* enzymes at the maximal enzyme load (Fig. 2). When half of the enzyme load was omitted from the reaction medium, the glucan conversion decreased to 75 ± 1 %. Restoring the original EG activity in the reaction media with the *L. sulphureus* and the *P. ostreatus* extracts resulted in glucan conversion levels of 87 ± 2 and 75 ± 2 %, respectively (Fig. 2). In all cases, cellobiose was not detected in the reaction media, suggesting a complete ability of the enzyme mixtures to produce glucose as the main product. These data indicate that the *L. sulphureus* extract restored the glucan conversion efficiency to values higher than those observed for the full *T. reesei* enzyme load. For the case of the *P. ostreatus* extract, the addition of a protein load to restore the original EG activity did not affect the final conversion efficiency.

The initial rates of glucan hydrolysis are shown in Table 2. When half of the original EG activity originated from the *L. sulphureus* and *P. ostreatus* extracts, the initial cellulose hydrolysis rates were higher than those detected in the experiment employing 50 % of *T. reesei* load, reaching similar rate values observed when the full *T. reesei* enzyme loading was used. This result was obtained even though the EG, CBH and β-glucosidase activity proportions varied significantly among the tests (See Fig. 2 legend). The initial glucan conversion rate results (Table 2) suggested that the assays using wood decay fungal proteins exhibited an efficient initial glucan conversion to glucose despite the low CBH activities in the reaction media. However, the maximal glucan conversion values were increased only when the *L. sulphureus* extract was used.

The xylan conversions in the experiments with the *L. sulphureus* and *P. ostreatus* extracts were higher than those observed with the maximal *T. reesei* enzyme load (Fig. 2). The data are consistent with the highest xylanase activity detected in the wood decay fungal extracts (Table 1). These data suggest that xylan-rich substrates, such as those resulting from the alkaline-sulfite pretreatment, would require xylanase-enriched enzymatic cocktails, such as those produced by the wood decay fungi, to produce an efficient xylan conversion.

The data collectively indicated that the crude protein extracts prepared from the wood decay fungi were important sources of supplementary enzymes for polysaccharides hydrolysis of alkaline-pretreated materials. However, a straight explanation for the high efficiencies of the glucan and xylan hydrolyses induced by the wood decay fungal protein extracts, even when CBH activity was low in the reaction media, was complicated by the numerous proteins present in the extracts (Table 1; Additional file 2: Table S2). For this reason, an LC–MS/MS-based analysis was performed to examine the protein compositions of the fungal protein extracts used in these hydrolysis experiments (Additional file 2: Table S2).

### Secretome analyses of wood decay fungal cultures

**Fig. 3:**
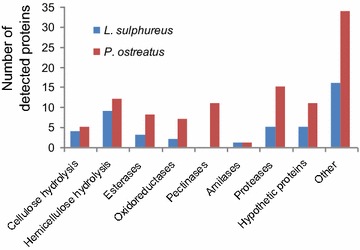
Protein diversity found in the *L. sulphureus* and *P. ostreatus* culture extracts classified according to their putative biological function. The total number of proteins identified in each extract was 45 for *L. sulphureus* and 104 for *P. ostreatus*

LC/MS/MS-identified peptides were compared with predicted proteins reported in the JGI and NCBI databases for *L. sulphureus* and *P. ostreatus* (Additional file 2: Table S2). Overall, 45 and 104 different protein matches were obtained for *L. sulphureus* and *P. ostreatus*, respectively (Fig. 3). The physical–chemical properties of the reported proteins are theoretical values based on the ProtParam tool (http://web.expasy.org/protparam; Additional file 2: Table S2). Of the proteins identified for *P. ostreatus*, 43 were considered relevant for lignocellulose degradation based on their putative functions. They included 5 cellulases, 12 hemicellulases, 8 esterases, 7 oxidoreductases and 11 pectinases. Conversely, the *L. sulphureus* extract exhibited lower protein diversity, with 4 cellulases, 9 hemicellulases, 3 esterases and 2 oxidoreductases. Relevant carbohydrate-active enzymes (CAZymes) were detected, which may also explain the performance of the studied protein mixtures and may show potential as new enzymes in enzymatic cocktails for biomass conversion.

The three main enzyme groups involved in cellulose hydrolysis (EG, CBH and β-glucosidases) were identified in both wood decay fungal extracts (Additional file 2: Table S2). CBHs are associated with efficient cellulose hydrolysis [4]. They are usually associated with a CBM, and these enzymes exhibit processivity over cellulose chains, acting from both ends of the polymer. Although low CBH activities were detected in the extracts (Table 1), CBH protein matches were identified in *L. sulphureus* (1 protein) and *P. ostreatus* (3 proteins) (Additional file 2: Table S2). *P. ostreatus* CBHs were classified into the GH7 family (2 proteins, one of which associated with CBM1) and the GH6 family (1 protein associated with CBM1). The *L. sulphureus* genome differs from those of several brown-rot fungi that lack CBH-encoding genes [16, 18]. When present in brown-rot fungi, GH7-CBH often lacks CBMs [18]. This was the case for the GH7-CBH detected in the *L. sulphureus* extract.

EGs from the GH5 family were identified in both wood decay fungal extracts. In the *P. ostreatus* extract, the GH5-EG was associated with CBM1 whereas *L. sulphureus* produced a GH5-EG without a CBM. Multiple EGs from white- (*Volvariella volvacea* [38]) and brown-rot fungi (*Gloeophyllum trabeum* [20] and *Fomitopsis palustris* [19]) were previously reported as processive EGs. A processive EG may explain the high hydrolysis efficiencies of enzyme mixtures lacking high CBH activities. However, structural properties vary among processive EGs [4], suggesting that specific assays are necessary to confirm the processivity of the evaluated enzymes. For example, a control experiment was performed using a conventional (non-processive) EG that was purified from *T. emersonii* [39] (Table 2) and mixed with the *T. reesei* enzymes. Replacing 50 % of the EG activity from the *T. reesei* enzymes with the EG enzymes from *T. emersonii* resulted in glucan and xylan conversions (72 ± 3 and 68 ± 3 %, respectively, after 72 h of hydrolysis) that were similar or slightly lower than those obtained with 50 % of the original load of *T. reesei* enzyme load (75 ± 1 and 73 ± 1 %, respectively, after 72 h of hydrolysis). The initial hydrolysis rates were also unaffected by the *T. emersonii* EGs (Table 2). These results indicated that, unlike the observations with the *L. sulphureus* and *P. ostreatus* extracts, the *T. emersonii* EG was unable to restore the hydrolysis efficiency obtained with the original *T. reesei* enzyme load.

A single EG from the GH45 family with high spectral counts was exclusively found in the *L. sulphureus* extract (Additional file 2: Table S2). Sequence protein similarities indicate that members of the GH45 family share homology with plant expansins and fungal swollenins that are secreted by ascomycetes, such as *T. reesei* [9, 40]. Both GH-45 and CBM1-swollenins have been associated with positive synergistic effects on cellulose hydrolysis. For example, the GH45-EG from *P. chrysosporium* acts synergistically with CBHs to increase the hydrolysis efficiency of phosphoric acid-swollen cellulose and crystalline cellulose [41]. Expansins and swollenins have been described as small proteins that have no hydrolytic activity towards cellulosic substrates. However, a recent study uncovered hydrolytic activity by a purified SWOI, and suggested that the protein behaves similar to EGs and CBHs [42].

**Fig. 4:**
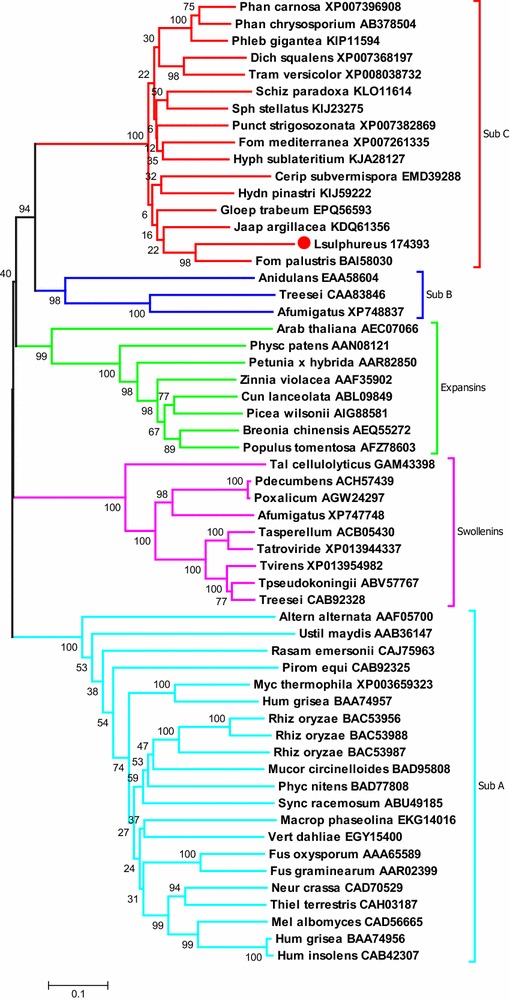
The phylogenetic tree of the amino acid sequences, including proteins from the GH45-EGs, swollenins and expansins. The EG45-EGs expressed by *L. sulphureus* (highlighted in *red*) was included in subfamily C together with other GH45-EGs from the white- and brown-rot basidiomycetes

To provide insights in the role of the GH45 from basidiomycetes, we performed an evolutionary and phylogenetic analysis that included several GH45-EGs, fungal swollenin and plant expansin sequences (Fig. 4). The phylogenetic tree analysis revealed that GH45-EG from *L. sulphureus* associated with a single cluster referred to as GH45-subfamily C that included other basidiomycete GH45s, which is consistent with the previous study that utilized an alternative method [41]. The GH45-subfamily C was more closely related to GH45-subfamily B (found in some ascomycetes) than to the swollenins.

**Fig. 5:**
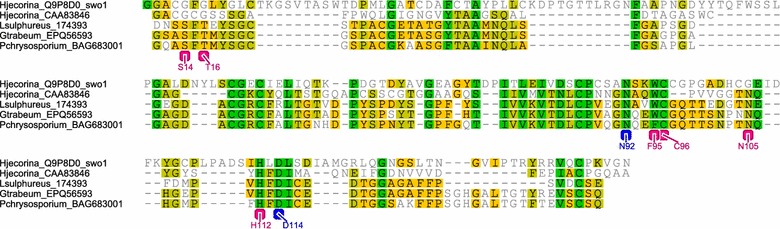
Comparative pairwise alignment of the expansin/GH45 domain of *L. sulphureus* with two basidiomycetes GH45-EGs (*G. trabeum* and *P. chrysosporium*), with one ascomycete GH45-EG (*H. jecorina*) and with one CBM1-swollenin from *H. jecorina*. The biochemically characterized GH45-EG from *P. chrysosporium* is indicated in *blue* for the catalytic amino acids (N92 and D114) and in *pink* for the neighboring amino acids involved in the proton transfer network

The amino acid sequence for the *L. sulphureus* GH45-EG was compared to other wood decay GH45-EGs, a reference *H. jecorina* GH45-EG, and the *H. jecorina* CBM1-swollenin [9]. A comparative pairwise alignment of *L. sulphureus* expansin/GH45 domain with those of *G. trabeum* and *P. chrysosporium* showed higher sequence identities (68.4 and 60.4 %, respectively) than the identities observed for the *H. jecorina* proteins (32.1 and 22.8 % for GH45-EG and CBM1-swollenin, respectively) (Fig. 5). The biochemically characterized *P. chrysosporium* GH45-EG contains two catalytic amino acids (asparagine-N^92^ and aspartic acid-D^114^) [43] that are conserved in all of the evaluated sequences, including the CBM1-swollenin from *H. jecorina.* Although multiple protein alignments showed conserved regions in both the swollenins and GH45-EGs, the swollenins contained some insertions between the “catalytic” amino acids (Fig. 5).

The catalytic mechanism proposed for *P. chrysosporium* GH45-EG [43] suggests that multiple neighboring amino acids between the catalytic amino acids are involved in the proton transfer network, enabling the hydrolysis of the β-1,4-glycoside bonds. The wood decay GH45-EG comparisons showed that these neighboring amino acids were highly conserved in the GH45-EGs but were absent in the CBM1-swollenin from *H. jecorina*. Together, these observations suggest that the *L. sulphureus* GH45-EG may provide a possible explanation for the high hydrolysis efficiency of this fungal extract in mixtures with commercial enzymes, even though with low CBH loads.

**Fig. 6:**
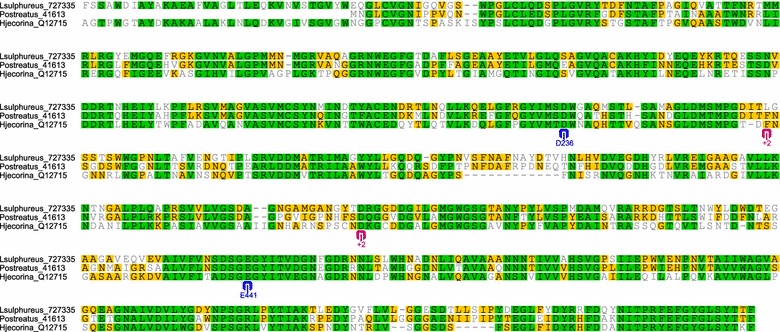
Comparative pairwise alignment of the GH3 domain of *Cel3A* from *H.* jecorina with the L*. sulphureus* and *P. ostreatus* β- glucosidases. For the biochemically characterized GH3-Cel3A from *H. jecorina,* the catalytic amino acids (D236 and E441) are indicated in blue and the +2 subsite (F260 and D370) which is responsible for the its atypically high activity on cellotriose and cellotetraose, is indicated in *pink*

The presence of β-glucosidases was also noteworthy in the wood decay extracts (Additional file 2: Table S2). β-glucosidase overloading was a relevant factor in the enzyme mixtures that included the *L. sulphureus* and *P. ostreatus* extracts (Fig. 2). Only one GH3-β-glucosidase was detected in each wood decay fungal extract. A comparative pairwise alignment of the GH3 domain of the Cel3a from *H. jecorina* with that of the wood decay fungi β-glucosidases is shown in Fig. 6. The sequence identities were 51.8 and 50.2 % for *L. sulphureus* and *P. ostreatus*, respectively. The active site of the *H. jecorina* GH3-*Cel3A* was selected for comparisons because this enzyme was more active on cellotriose and cellotetraose than on cellobiose, suggesting a high processing capacity for oligosaccharides [44]. Its active site contains aspartic acid-D^236^ and glutamic acid-E^441^ residues. Both amino acids are conserved in the *L. sulphureus* and *P. ostreatus* GH3-β-glucosidases. The high oligosaccharide processing ability of the *H. jecorina* GH3-*Cel3A* was attributed to the +2 subsite, which comprised phenylalanine-F^260^ and aspartic acid-D^370^. This subsite was also observed in the wood decay GH3-β-glucosidases, though the phenylalanine-F^260^ was substituted by leucin-L^260^ in *L. sulphureus*. This finding is relevant because the efficient actions of GH3 on oligosaccharides allow overloading of this unusual GH3-β-glucosidase in the wood decay extracts to compensate for the low CBH activities.

Xylanase activities were also overloaded in the sugarcane bagasse hydrolysis experiments (Fig. 2). Proteomic studies indicated that endo-xylanases from the GH10 family were identified in the extracts from *P. ostreatus* (1 protein) and *L. sulphureus* (2 proteins) (Additional file 2: Table S2). Additional hemicellulases, including arabinofuranosidases, β-xylosidases and acetyl xylan esterases were identified in both protein extracts. The overloading of the xylanase activities and the diversity of the hemicellulases in the wood decay extracts may explain the more efficient xylan conversion in the hydrolysis experiments. The efficient xylan hydrolysis may have provided a changing substrate during the hydrolysis reaction that was progressively depleted in xylan, resulting in enhanced accessibility of cellulases to the cellulose substrate [8, 45].

LPMOs (AA9 in CAZy) can also enhance cellulase efficiency by oxidatively disrupting cellulose chains [4, 13]. Despite LPMOs were predicted based on the *P. ostreatus* and *L. sulphureus* genome analyses (24 and 2 predicted proteins, respectively), these proteins were not detected in the fungal extracts under evaluation. It is possible that during sample preparation, the previous concentration and desalting of the crude extracts against the 30 kDa-cut-off membranes could result in the loss of these low molar mass proteins. Alternatively, they may have remained adsorbed to the culture substrate in the case of sugarcane bagasse used for culturing *P. ostreatus*. AA9s were also not detected in secretome studies examining *P. ostreatus* grown on lignified substrates [28]. In contrast, other oxidoreductases, including laccases (5 proteins from the AA1 family), glyoxal oxidases (1 protein from the AA5 family) and DYP-type peroxidases were detected in the *P. ostreatus* extract (Additional file 2: Table S2) in agreement with this previous study [28]. Laccases have potential roles in the degradation/polymerization of the lignin fragments released during the course of enzymatic hydrolysis of the polysaccharides fraction. Recent reports highlight the role of laccases in avoiding the inhibitory coupling of these lignin fragments with the hydrolytic enzymes, thus enhancing hydrolysis efficiency [31].

## Conclusions

The extracellular proteins produced by white- and brown-rot fungi were useful supplements to commercial enzymes cocktails (produced by ascomycetes) during the saccharification of pretreated sugarcane bagasse. Proteomic studies revealed four protein families (GH5-EGs, GH45-EGs, GH3-β-glucosidases and GH10-xylanases) that showed promise towards improving current enzyme cocktails and enhancing alkaline-pretreated substrate saccharification. GH3-β-glucosidases from the wood decay fungi were similar to a GH3-β-glucosidase from *H. jecorina* that is highly active on oligosaccharides, suggesting a candidate enzyme to replace some of the CBHs used in the current enzyme cocktails. Other discovered enzymes with potential for using in lignocellulose hydrolysis included GH5-EGs, GH45-EGs and GH10-xylanases.

## Methods

### Microorganisms, culture conditions and enzymatic activity determinations

The white-rot fungus *P. ostreatus* (CCIBT-2347) and the brown-rot fungus *L. sulphureus* (ATCC 52600) were maintained on 20 g/L malt-extract agar slants containing a slice of wood at 4 °C. To prepare the inoculum, 200 mL of liquid medium containing potato extract broth (24 g/L) and yeast extract (7 g/L) was inoculated with 20 discs (8 mm diameter) of precultured solid medium with either *L. sulphureus* or *P. ostreatus*. The liquid culture was maintained without shaking for 14 days at 27 °C. The resulting mycelial mat was filtered and washed with 300 ml of sterile water. Mycelia obtained from several culture flasks were blended with 100 ml of sterile water in 3 cycles of 15 s. The mycelial suspension was used to inoculate Erlenmeyer flasks (125 mL) containing 40 mL of liquid medium at 500 µg mycelium (oven-dry basis)/mL. The liquid medium for the enzyme production tests consisted of 1.0 g/L NH_4_NO_3_, 0.5 g/L MgSO_4_, 0.2 g/L Na_2_HPO_4_, 0.8 g/L KH_2_PO_4_, and 4.0 g/L yeast extract and contained a specific carbon source for each broth as follows: (a) 20 g/L microcrystalline cellulose (Avicel, Fluka PH-101); (b) 20 g/L carboxymethyl cellulose (SIGMA C4888); (c) 20 g/L lactose; (d) 40 g/L milled sugarcane bagasse; (e) 20 g/L lactose plus 40 g/L sugarcane bagasse; (f) 20 g/L carboxymethyl cellulose plus 40 g/L sugarcane bagasse, and (g) 20 g/L lactose plus 20 g/L carboxymethyl cellulose plus 40 g/L sugarcane bagasse. Inoculated flasks were maintained without shaking at 27 °C. The cultures were periodically sampled between the 4th and 50th day by filtering the content of one culture flask from each media group. Each filtered culture broth was assayed for endoglucanase activity [34].

For preparative enzyme production, *L. sulphureus* was grown for 24 days in medium containing 20 g/L carboxymethyl cellulose as described before. Filtrates were combined and mixed with (NH4)_2_SO_4_ to reach 80 % saturation to precipitate the proteins from the culture broth (the phenol–sulfuric acid test returned a negative result for the precipitate, indicating the absence of CMC). Precipitate proteins were centrifuged at 3400×*g* at 4 °C for 15 min. The pellet was desalted by dissolution in 25 mL of distilled water and ultra-filtration in a 30-kDa cut-off Amicon Ultra centrifugal filter (Millipore, Ireland). EG activity (used as a titer for the recovery procedures) recovered in these procedures was limited to 15 % of the initial values.

For the *P. ostreatus* enzymes, 42-day-old cultures grown in media containing 40 g/L sugarcane bagasse were filtered through glass filters. Filtrates were concentrated by ultra-filtration in a 30-kDa cut-off Amicon Ultra centrifugal filter (Millipore, Ireland). The recovery yield of the EG activities in the retained fraction was 60 %. The concentrated extract was freeze-dried to yield the *P. ostreatus* protein extract used in subsequent experiments.

The protein concentrates were assessed for their protein content, EGs and filter paper activities [34]. The CBH activity was determined using Avicel (Fluka, PH-101) as the substrate [46]. β-glucosidase and β-xylosidase activities were assayed by measuring the quantity of *p*-nitrophenol released from the corresponding *p*-nitrophenyl-glycosides [47]. Endo-xylanase activity was determined using birch wood xylan (SIGMA X-0502) as the substrate [48].

### Pretreatment of sugarcane bagasse and chemical characterization of biomass samples

Sugarcane bagasse was collected in a sugar and ethanol mill after sugarcane processing for sucrose extraction. The entire biomass was air dried and stored in dry conditions. A pretreatment procedure was performed using a two-step lab-scale process that simulated the chemi-thermomechanical pulping [35].

The chemical compositions of the untreated and pretreated sugarcane bagasse samples were determined using ethanol-extracted materials as previously described [35]. This procedure was performed in triplicate and the average values and standard deviations are reported in the text.

### Enzymatic hydrolysis of the pretreated sugarcane bagasse

The enzymatic hydrolysis of the pretreated sugarcane bagasse utilized three different commercial enzymes and crude protein extracts prepared from the *P. ostreatus* or *L. sulphureus* cultures. The crude commercial enzymes included cellulases from *T. reesei* (SIGMA C2730), β-glucosidases from *A. niger* (SIGMA C6105), and purified EGs from *Talaromyces emersonii* (Megazyme 30602). Enzyme loadings varied between experiments as indicated in the “Results and discussion” section. The *A. niger*-derived commercial β-glucosidase (SIGMA C6105) was used at 15 IU/g of substrate to supplement the reaction media in all experiments. All experiments were performed on a micro-scale, where 20 mg of pretreated sugarcane bagasse was digested in a 2-mL microcentrifuge tube with a 1-mL enzyme solution containing 50 mM sodium-acetate buffer (pH 5.0) and 0.01 % sodium azide. The tubes were incubated at 45 °C under reciprocal agitation at 120 cycles per min. The reaction was stopped at defined points between 4 and 72 h by cooling in ice, followed by centrifugation at 3400×*g* for 15 min at 4 °C. Twenty microliters of the liquid hydrolysate were diluted with water to assess the presence of monomeric sugars by HPLC. The remaining content was re-incubated at 45 °C to continue the hydrolysis reaction. The diluted hydrolysates were assayed for glucose and xylose contents using a BIO-RAD HPX87H column at 45 °C with 5 mM sulfuric acid at 0.6 mL/min for the elutions. Sugars were detected using a temperature-controlled RI detector in a HPLC system (2414 model, Waters, Milford, MA). Cellulose and xylan enzymatic conversions were calculated based on the levels of glucose and xylose released from the glucan and xylan contained in the pretreated sugarcane. Three replicate flasks were incubated for each reaction condition. Variations between hydrolysis replicates are presented as the standard deviation values. Average conversion levels and initial hydrolysis rate values were compared using Tukey’s HSD (honest significant difference).

### Protein digestion and liquid chromatography–tandem mass spectrometry (LC–MS/MS)

The protein digestion for the mass spectrometry-based analyses were performed in two steps over 2 days [49]. On day one, 7 gel-bands per lane were excised from an SDS-PAGE of enzymatic extracts produced by *L. sulphureus* and *P. ostreatus*. The SDS and coomassie stain were removed using 500 µL of a destaining solution (10 % glacial acetic acid and 10 % ethanol) for 2 h, and the bands were dehydrated for 5 min with 200 µL acetonitrile, reduced for 30 min with 30 µL dithiothreitol (DTT) solution (10 mmol/L) and alkylated for 30 min with 30 µL iodoacetoamide (IAA) solution (50 mmol/L). The bands were then washed with 100 mmol/L ammonium bicarbonate for 10 min. Another dehydration step was performed with acetonitrile, followed by rehydration with 50 mmol/L sodium bicarbonate. Protein digestion was performed with 30 µL of trypsin (1 mg/mL) in 50 mmol/L ammonium bicarbonate at 37 °C overnight. On day 2, 20 µL of an extraction solution composed of 5 % (v/v) formic acid was added to each micro centrifuge tube and incubated for 10 min at room temperature. After a rapid centrifugation step, the supernatant was collected and transferred to another microcentrifuge tube. Afterward, 30 µL of a second extraction solution composed of 5 % (v/v) formic acid in 50 % (v/v) acetonitrile were added to the first pellet. After 10 min, the supernatant was collected and transferred to a tube containing the extract from the previous step. The last procedure was repeated one time. Finally, the samples were evaporated under vacuum to approximately 1 µL. The samples were stored at −20 °C until further analysis by LC–MS/MS.

Each sample was mixed with 12 µL of 0.1 % (v/v) formic acid, and 4.5 µL of the peptide mixture were injected into the LC–MS/MS chromatograph (RP-nanoUPLC, nanoAcquity, Waters, Milford, MA). Peptide separations were performed on a C18 column (100 nm × 100 mm) previously equilibrated with a 0.1 % (v/v) formic acid buffer. The elution gradient ranged from 2 to 90 % (v/v) acetonitrile in 0.1 % (v/v) formic acid at 0.6 µL/min. Eluted peptides were analyzed in a quadrupole time of flight (Q-TOF) spectrometer (Ultima Mass Spectrometer, Waters Milford, MA). The instrument was operated in the “top three-MS and MS/MS mode” (Ultima Mass Spectrometer, Waters software). The spectra were acquired using the MassLynx v.4.1 software (Waters, Milford, MA, USA), and the raw data were converted to “peak list format (mgf)” using the Mascot Distiller software v.2.3.02, 2009 (Matrix Science Ltd., London, UK). The results were processed by the Mascot v.2.3.02 engine software (Matrix Science Ltd.) against the Joint Genome Institute’s genome database for the *P. ostreatus* and *L. sulphureus* strains (12,330 sequences; 5,136,290 residues and 13,774 sequences; 5,395,426 residues, respectively). The following parameters were used in this process: carbamidomethylation as a fixed modification, oxidation of methionine as a variable modification, one trypsin cleavage error and a maximum allowable peptide mass error 0.1 Da. Peptides with five or more amino acids and scores indicating a low probability for random event (*p* < 0.05) were selected as peptide cleavage products (an indication that they were part of a protein). The peptide was considered relevant when it differed from another peptide by at least one amino acid or when differs in covalent modifications (including elongations of N- or C-terminal). The resulting Mascot data were analyzed for protein identification using Scaffold 3.5.1 (Proteome Software, Portland, OR). The defined parameters were as follows: a minimum protein probability of 80 %, a minimum peptide probability of 90 % and a uniquely different minimum peptide of 1. We accepted proteins with scores up to 10 % FDR (false discovery rate) for a protein and 5 % FDR for a peptide.

### Identification of protein sequences, annotation and phylogenetic analyses

All protein sequences were downloaded from JGI (http://www.jgi.doe.gov). Protein sequence identities were based on pairwise alignments generated using the multiple sequence comparison by log-expectation (MUSCLE) software through the Geneious v.4.5.5 software [50]. The protein domains were established using the InterPro v.54 database (http://www.ebi.ac.uk/interpro/). For phylogenetic analyses, multiple alignments of the complete sequences were produced using the MUSCLE database (http://www.ebi.ac.uk/Tools/msa/muscle/) with default parameters [51]. Phylogenetic trees were constructed employing a neighbor-joining method [52], with the *p* distance matrix model and pairwise deletion, and with 1000 bootstrap replicates, using the MEGA 6.06 software [53].

## Additional files

10.1186/s13068-016-0525-y Chemical composition and processing yield of sugarcane bagasse pretreated by an alkaline-sulfite process using 5 % NaOH and 10 % Na_2_SO_3_.
10.1186/s13068-016-0525-y Proteins detected in the crude extracts recovered from *Laetiporius sulphureus* cultures using CMC as carbon source are listed in the first sheet of the excel spreadsheets; Proteins detected in the crude extracts recovered from *Pleurotus ostreatus* cultures using sugarcane bagasse as carbon source are listed in the second sheet of the excel spreadsheets.

## Abbreviations

GHs: glycoside hydrolases
CBH: cellobiohydrolases
EG: endoglucanases
CBM: cellulose binding module
SWO: swollenin
LPMOs: lytic polysaccharide mono-oxygenases
CDHs: cellobiose dehydrogenases
CMC: carboxymethyl cellulose
PNPG: 4-nitrophenyl β-d-glucopyranoside
PNPX: 4-nitrophenyl β-d-xylopyranoside
FPU: filter paper units
