# Supplementary material for: Exploring glycoside hydrolases and accessory proteins from wood decay fungi to enhance sugarcane bagasse saccharification
Source: Biotechnol Biofuels. 2016 May 23;9:110. doi: 10.1186/s13068-016-0525-y (PMC4877993; doi:10.1186/s13068-016-0525-y)
Supplement: Supplementary file 1 — 10.1186/s13068-016-0525-y Chemical composition and processing yield of sugarcane bagasse pretreated by an alkaline-sulfite process using 5 % NaOH and 10 % Na2SO3. [file 13068_2016_525_MOESM1_ESM.docx]

**Table S1**. Chemical composition and processing yield of sugarcane bagasse pretreated by an alkaline-sulfite process employing 5% NaOH and 10% Na_2_SO_3_

| Sugar cane bagasse sample | Chemical composition  (g/100 g of the lignocellulosic material) | | | Processing yield (%) | Mass balance of bagasse components  (g/100 g of original bagasse) | | |
| --- | --- | --- | --- | --- | --- | --- | --- |
|  | Glucan | Hemicellulose | Lignin |  | Glucan | Hemicellulose | Lignin |
| untreated | 42 ± 2 | 26 ± 1 | 24.0 ± 0.1 | 100 | 42 ± 2 | 26 ± 1 | 24.0 ± 0.1 |
| alkaline-sulfite pretreated | 54.7 ± 0.6 | 20.3 ± 0.1 | 14.7 ± 0.5 | 74.9 | 41.0 ± 0.4 | 15.2 ± 0.1 | 11.0 ± 0.4 |
